# A Genome Wide Meta-Analysis Study for Identification of Common Variation Associated with Breast Cancer Prognosis

**DOI:** 10.1371/journal.pone.0101488

**Published:** 2014-12-19

**Authors:** Sajjad Rafiq, Sofia Khan, William Tapper, Andrew Collins, Rosanna Upstill-Goddard, Susan Gerty, Carl Blomqvist, Kristiina Aittomäki, Fergus J. Couch, Jianjun Liu, Heli Nevanlinna, Diana Eccles

**Affiliations:** 1 Genetic Epidemiology and Bioinformatics Research Group, Human Genetics, Faculty of Medicine, University of Southampton, Southampton General Hospital, Hants, United Kingdom; 2 Clinical Trials Unit, Faculty of Medicine, University of Southampton, Hants, United Kingdom; 3 Department of Laboratory Medicine and Pathology, Mayo Clinic, Rochester, Minnesota, United States of America; 4 Department of Obstetrics and Gynaecology, University of Helsinki and Helsinki University Central Hospital, Helsinki, Finland; 5 Department of Oncology, Helsinki University Central Hospital, Helsinki, Finland; 6 Department of Clinical Genetics, Helsinki University Central Hospital, Helsinki, Finland; 7 Human Genetics, Genome Institute of Singapore, Singapore; 8 Cancer Sciences Division, University of Southampton, School of Medicine, Southampton General Hospital, Hants, United Kingdom; MOE Key Laboratory of Environment and Health, School of Public Health, Tongji Medical College, Huazhong University of Science and Technology, China

## Abstract

**Objective:**

Genome wide association studies (GWAs) of breast cancer mortality have identified few potential associations. The concordance between these studies is unclear. In this study, we used a meta-analysis of two prognostic GWAs and a replication cohort to identify the strongest associations and to evaluate the loci suggested in previous studies. We attempt to identify those SNPs which could impact overall survival irrespective of the age of onset.

**Methods:**

To facilitate the meta-analysis and to refine the association signals, SNPs were imputed using data from the 1000 genomes project. Cox-proportional hazard models were used to estimate hazard ratios (HR) in 536 patients from the POSH cohort (Prospective study of Outcomes in Sporadic versus Hereditary breast cancer) and 805 patients from the HEBCS cohort (Helsinki Breast Cancer Study). These hazard ratios were combined using a Mantel-Haenszel fixed effects meta-analysis and a p-value threshold of 5×10^−8^ was used to determine significance. Replication was performed in 1523 additional patients from the POSH study.

**Results:**

Although no SNPs achieved genome wide significance, three SNPs have significant association in the replication cohort and combined p-values less than 5.6×10^−6^. These SNPs are; rs421379 which is 556 kb upstream of *ARRDC3* (HR = 1.49, 95% confidence interval (CI) = 1.27–1.75, P = 1.1×10^−6^), rs12358475 which is between *ECHDC3* and *PROSER2* (HR = 0.75, CI = 0.67–0.85, P = 1.8×10^−6^), and rs1728400 which is between *LINC00917* and *FOXF1*.

**Conclusions:**

In a genome wide meta-analysis of two independent cohorts from UK and Finland, we identified potential associations at three distinct loci. Phenotypic heterogeneity and relatively small sample sizes may explain the lack of genome wide significant findings. However, the replication at three SNPs in the validation cohort shows promise for future studies in larger cohorts. We did not find strong evidence for concordance between the few associations highlighted by previous GWAs of breast cancer survival and this study.

## Introduction

Although the incidence of breast cancer has been relatively stable since 2003, at 157 new cases per 100,000, it remains the most common cancer in the UK and accounts for 31% of new cancer cases in women. The latest age-standardised survival rate for breast cancer in England is predicted to be 85% at 5 years, falling to 65% at 20 years [1]. Traditionally prognostic information is derived from tumour phenotypic characteristics including tumour size, stage, and grade. These tumour phenotypes and cancer cell surface receptors such as oestrogen receptor (ER) and human epidermal growth factor receptor 2 (HER2) are also used to guide treatment. Although the breast cancer survival rate has improved, the response to treatment and longevity of patients is often unpredictable even between those with similar tumours and general health. More recently tumour genomic profiling experiments have suggested cancer molecular signatures may give more accurate prognostic information [2]–[4]. These signatures may predict outcome better than conventional histopathology based risk algorithms but are not in routine clinical use [5].

Familial studies suggest a genetic component for breast cancer prognosis [6], [7]. The familial contribution to prognosis may arise as a result of the background genotype affecting acquired tumour characteristics which influence prognosis. Indeed high penetrance predisposition genes which lead to the consistent development of specific breast tumour sub-types have been identified [8], [9]. Low penetrance risk SNPs tend to be associated with either ER positive or ER negative breast cancer but often not both [10]–[14]. In addition there may be pharmacogenomic effects of background genotype on response to cancer treatment. It is anticipated that genome wide association studies (GWAs) with sufficient sample size and genetic coverage may lead to novel insights into common inherited genetic variants which influence prognosis.

In the past few years several GWAs of breast cancer survival have been reported. These studies have had limited success and none of them have identified variants that are associated at genome wide levels of significance [15]–[19]. While small sample sizes are likely to be of one of the main factors responsible for the modest levels of significance and lack of concordance between the GWAs; small effect sizes, incomplete genetic coverage, and phenotypic heterogeneity could also contribute and need to be addressed.

In this study, we used a meta-analysis to combine evidence from two GWAs consisting of 536 patients from the POSH cohort (Prospective study of Outcomes in Sporadic versus Hereditary breast cancer) and 805 patients from the HEBCS cohort (Helsinki Breast Cancer Study). A further 1523 patients from the POSH cohort were used to validate the most significant SNPs. With a combined sample size of 2864 participants, this analysis has 81% power to detect effects of modest sizes (HR≥1.25, p = 0.05) and with relatively rare SNPs (MAF = 10%). The cohorts used in this analysis have a high incidence of breast cancer related mortality and well documented tumour and treatment data which make them ideal for the purpose of exploring genetic factors influencing prognosis. In addition, these cohorts are similar in terms of their patient recruitment from regional medical centres, duration of prospective follow-up, and documentation of breast cancer related mortality.

## Materials and Methods

All participants from POSH and Helsinki gave written informed consent, all were female. The POSH study received approval from the South and West Multi-centre Research Ethics Committee (MREC 00/6/69). The Helsinki breast cancer study received approval from the Ethical Committee of the Departments of Oncology and Obstetrics and Gynaecology, Helsinki University Central Hospital.

### Breast cancer patients and genotyping

Breast cancer cases were selected from the POSH study and the Helsinki breast cancer family Study (HEBCS). POSH study participants were diagnosed with invasive breast cancer and were aged forty or younger at diagnosis, the mean age at diagnosis in this cohort is 36 years. Recruitments to the POSH cohort were made between January 2000 and January 2008 from oncology clinics across the UK and the majority (98%) of patients presented symptomatically. The recruitment, data collection and follow up procedures for the POSH study participants are described in detail elsewhere [20].

The HEBCS samples were collected in Helsinki, Finland and are representative of breast cancer case series at the recruitment centre during the collection periods (unselected sporadic and familial cases collected between 1997 and 2004). All of the cases used in the meta-analysis had histopathological and survival data. Detailed information on the patient series and data collection has previously been published [21]. The mean age at diagnosis was 56.8 years.

### Stage 1 discovery dataset

In stage-1, 574 participants from the POSH study were selected for the discovery phase of the analysis aimed at hypothesis generation [20]. In keeping with a recent GWAS which identified five new breast cancer susceptibility loci by enriching cases by recruiting individuals with family history of breast cancer [22], sample selection for stage-1 utilised an “extreme phenotype” approach, this included selection of triple negative cases genotyped in a collaboration aimed at risk associated SNPs in triple negative breast cancer [11] and a second group enriched for exceptionally short survival genotyped as described previously [23]. We observed 236 breast cancer specific deaths in the POSH discovery set patients.

In HEBCS, 805 cases were selected from the patient series described earlier [22], including 423 unselected cases collected between years 1997 and 2000 as well as 140 cases collected between years 2001 and 2004, with 242 additional familial cases. The GWAS series was specifically enriched for cases with reduced survival, in the form of distant metastasis or death at the time of the initiation of the study in 2008, resulting in 301 breast cancer specific deaths at the time of analysis.

### Stage-2 replication Samples

A further 1523 breast cancer patients from the POSH study [20] unselected for any survival differential were used for replication in stage-2. At stage 2, there were 293 breast cancer specific deaths.

### Genome wide genotyping

Genotyping of 574 POSH phase-1 breast cancer cases was conducted using the Illumina 660-Quad SNP array. Genotyping was conducted in two separate batches at two locations. The Mayo Clinic (Rochester, Minnesota, USA) genotyped 274 triple negative breast cancers (negative for ER, PR and HER2) [11]. The remaining 300 POSH patients were genotyped at the Genome Institute of Singapore (GIS), National University of Singapore; these were selected based on either short duration of breast cancer specific survival (<2 years) or long duration of breast cancer specific survival (>4 years). In order to ensure complete harmonisation of genotype calling, the intensity data from GIS and MAYO were combined and the genotyping module of Illumina’s Genome Studio software was used to generate genotypes. A GenCall threshold of 0.15 was selected and the HumanHap660 annotation file was used. Of the 300 samples genotyped in Singapore, 3 were excluded from analysis because they had sample call rates lower than 95%. No individuals among the two hundred and seventy four triple negative cohort genotyped at the Mayo clinic were excluded from analysis based on poor call rate. The genotyping accuracy for SNPs genotyped by GIS and Mayo were over 99%.

Genotyping of the HEBCS samples was conducted using the Illumina 550 platform as previously described [24]. SNP quality control (QC) measures were implemented using Plink. The initial sample size of 832 was reduced to 805, following quality control measures to remove patients with; unidentified affectation status and gender discordance (n = 6), familial relationships and poor SNP call rate (<95% n = 18), and missing phenotype information (n = 1). Genotypes were determined using the Genome Studio, a GenCall threshold of 0.15, and the HumanHap550-duo v3 annotation file.

Further quality control of the genotypic data from POSH and HEBCS was used to exclude rare SNPs with a MAF ≤0.01, and SNPs with significant deviation from Hardy-Weinberg equilibrium (HWE) p-value≤0.0001. To select SNPs for generation of pairwise identity by state (IBS) estimates, we used plink to perform genome wide linkage disequilibrium (LD)-based pruning with an r^2^ cut-off of 0.5 and a window of 50 SNPs. Multi-dimensional scaling (MDS) plots were generated on the basis of a square matrix of IBS values between all pairs of individuals. To act as a reference, individuals with known African, Asian, and Caucasian ancestry from HapMap were also used for the MDS analysis [25]. The MDS analysis excluded 35 cases from POSH and no cases from Helsinki whose genotypes did not concur with a European ancestry.

### Statistical Analysis

We used GenABEL [26] in R.2.14.0 environment to perform survival analysis using post-QC genome wide SNP data. Follow-up time was calculated as the difference between the date of diagnosis of breast cancer and the date of death due to breast cancer or the date of last follow-up if still alive and right-censored at 10 years. Distant disease free interval was calculated as the time from diagnosis to occurrence of metastasis. We excluded patients with contralateral or ipsilateral cancers for testing association with distant disease free interval. All the Cox-proportional hazard models were adjusted for ER-status. Kaplan-Meier plots were generated using STATA v11.0 and IBM SPSS statistics 19. Mantel-Haenszel Fixed effects meta-analysis was performed using the metan module in STATA v11.0 [27]. For multivariate models we used ER-status, metastasis stage (0 or 1), nodal stage (1 = no nodes positive, 2 = 1–3 nodes positive, 3 = more than 3 nodes positive) and tumour size (centimetres) as covariates.

Cochran’s Q-statistic and the resultant p-value was used to detect heterogeneity in association estimates between POSH and HEBCS. Genome wide meta-analysis was performed using MetABEL [28].

### Genome wide imputation and meta-analysis

We imputed genome wide SNP information in POSH and HEBCS based on European phase 1 and release version 3 haplotypes. The reference haplotypes are derived from the 1000 genomes project which is the most comprehensive catalogue of human genetic variation including SNP, Indels and CNVs. Quality control measures applied to imputed data included excluding SNPs with HWE p-value<1×10^−6^, MAF <5%; and genotyping call rate <90% and individuals call rate <90%. Genome wide survival analysis of imputed information was performed in R-2.14.0 using GenABEL. Meta-analysis of results from GenABEL was performed using MetABEL. For imputing data we used MACH (http://www.sph.umich.edu/csg/abecasis/MACH/index.html). We used VCFtools - v0.1.9.0 to generate plink format files from output files generated by MACH. The reference haplotypes for 1000 genomes project were downloaded from MACH software’s download page. We used Phase I version 3 European reference haplotypes for imputation analysis.

### Manhattan and Regional plots

Manhattan and QQ-plots were generated in R using the plot command. Regional plots were generated using LocusZoom [29].

### Sample size calculations

Sample size calculations were performed in R.2.14.2 using survSNP package. The event rate used for power calculations was 0.29 and a two-sided alpha of 0.05 was applied.

### Gene Expression variation by SNP

We used Genevar 3.2.0 to study variation in expression levels by SNP genotypes available from the MuTHER pilot project while using NCBI Build 36 Ensembl 54 as reference [30]. Twin pairs were divided into two groups of unrelated individuals. Expression data from Lymphoblastoid cell lines are reported here. In addition we used SNP and CNV annotation database (scandb) [31] that uses the lymphoblastoid cell line expression data derived from 90 HapMap CEU samples in trios [32].

### Prediction of transcription factor binding site changes

The putative changes on transcription factor binding sites caused by the variants were predicted *in silico* with MatInspector within Genomatix software suite v2.5 (Genomatix Software GmbH) [33].

## Results

### POSH stage-1 and HEBCS meta-analysis

**Figure 1:**
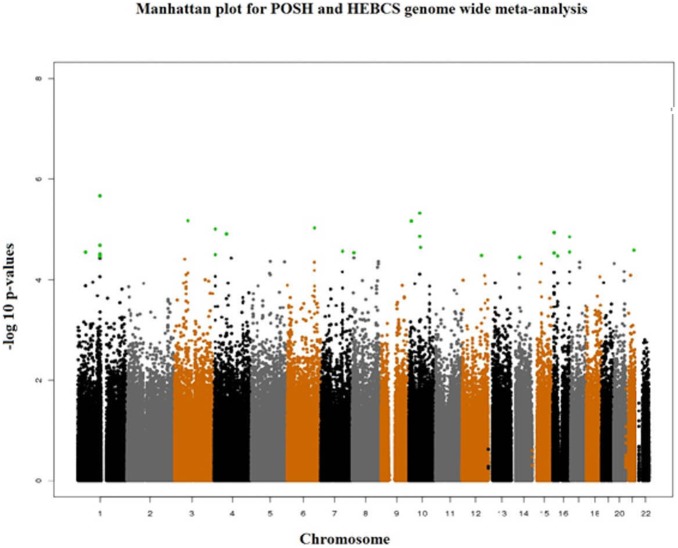
Manhattan plot of results from genome wide meta-analysis of POSH stage-1 and HEBCS hazard ratios and 95% confidence intervals. The 25 most associated SNPs are highlighted in green.

Genome wide genotype data were available from 536Caucasian participants of the POSH study and 805 Caucasian participants of the HEBCS study. A total of 475,141 SNPs with observed genotypes were available for meta-analysis in both the studies after excluding SNPs based on QC criteria. In stage-1 we used fixed-effects meta-analysis to pool hazard ratio estimates from the 536 POSH and 805 Helsinki breast cancer patients (Table 1). In the two study meta-analysis we found five SNPs which were associated at p-values lower than 9.9×10^−6^ (Table 2, Figure 1). The 25 most associated loci were selected for replication in POSH stage-2 patients. For loci with more than one SNP associated with survival, the most significant SNP and any other SNP(s) from the same locus which were not in high LD with the lead SNP (r^2^<0.6) were selected for follow up in stage-2 (Table 3).

**Table 1:** Clinical characteristics of Study participants from the discovery and replication sets.

Study	Number of breast cancer deaths	Total number of Breast cancer patients	Estrogen Receptor (ER) status- Negative (%)	Average age at Diagnosis (±SD)	Follow-up time in years (±SD)	N-stage	M-stage	T-stage
POSH stage-1 (Discovery)	236	536	370 (69.2%)	35.7 (3.8)	4.1 (2.0)	N0–248N1–262NA-26	M0–481M1–50NA-5	T1–227 T2–207 T3–20 T4–31 NA-51
HEBCS (Discovery)	301	805	230 (30.0%) NA-39	56.8 (12.4)	7.2 (2.9)	N0–338N1–446NA-21	M0–740M1–57NA-8	T1–390 T2–304 T3–50 T4–47 NA-14
POSH stage-2 (Replication)	221	1415	362 (23.7%)	35.8 (3.5)	5.2 (1.7)	N0–705N1–810NA-8	M0–1506M1–18NA-1	T1–692 T2–494 T3–49 T4–34 NA-254

HEBCS: Helsinki Breast Cancer Study; NA = not available, HER2 = Human Epidermal Growth Factor Receptor 2, N-stage = metastasis to lymph node, M-stage = metastasis.

**Table 2:** SNPs representing the 25 most significant associations in the discovery sets (after excluding SNPs in relative LD≥r^2^ of 0.60 and associated with less significant p-value with lead SNP at a locus) and their association estimates (adjusted for ER-status).

Lead SNP	Chr	Position	Alleles	MAF	POSH stage-1 pre meta-analysis HR(95% CI)	HEBCS premeta-analysis HR(95% CI)	POSH stage-1 and HEBCSmeta-analysisHR (95% ConfidenceIntervals)	POSH stage-1 and HEBCSmeta-analysisp-value	Genes
rs12026014	1	39060495	G/A	0.39	0.70 (0.57–0.86)	0.80 (0.68–0.95)	0.76 (0.69–0.84)	2.84×10^−5^	POU3F1: LOC400750
rs12735344	1	111559848	G/T	0.22	0.71 (0.58–0.89)	0.75 (0.61–0.88)	0.74 (0.67–0.82)	3.46×10^−5^	CCNT2P1
rs1149185	1	111546531	C/T	0.50	1.51 (1.26–1.80)	1.21 (1.03–1.42)	1.34 (1.22–1.46)	2.14×10^−6^	C1orf103: TMEM77
rs1578790	1	111575921	G/T	0.50	1.47 (1.23–1.77)	1.16 (0.99–1.36)	1.28 (1.16–1.52)	3.31×10^−5^	C1orf103: TMEM77
rs11723068	4	7797435	G/A	0.12	1.99 (1.51–2.64)	1.23 (0.99–1.54)	1.48 (1.18–1.99)	9.83×10^−6^	AFAP1
rs7441398	4	63653135	G/T	0.13	1.43 (1.14–1.79)	1.43 (1.14–1.79)	1.43 (1.16–1.85)	1.22×10^−5^	LPHN3: LOC644548
rs10457678	6	139122240	A/G	0.24	1.39 (1.15–1.72)	1.30 (1.10–1.55)	1.34 (1.21–1.47)	9.38×10^−6^	ECT2L
rs1525677	7	110302695	T/C	0.31	1.31 (1.09–1.58)	1.31 (1.10–1.56)	1.31 (1.18–1.44)	2.74×10^−5^	IMMP2L
rs13274039	8	8111659	A/G	0.28	1.15 (0.96–1.39)	1.41 (1.05–1.47)	1.29 (1.17–1.41)	2.92×10^−5^	FLJ10661: PRAGMIN
rs12358475	10	11848792	G/A	0.23	0.73 (0.59–0.90)	0.71 (0.58–0.86)	0.72 (0.65–0.80)	6.77×10^−6^	ECHDC3: C10orf47
rs2921923	10	55662089	A/G	0.49	1.40 (1.16–1.68)	1.26 (1.09–1.49)	1.32 (1.20–1.44)	4.73×10^−6^	PCDH15
rs10777864	12	97838685	A/C	0.40	0.79 (0.65–0.95)	0.74 (0.62–0.88)	0.76 (0.63–0.89)	3.28×10^−5^	RMST
rs1499384	14	43049048	A/G	0.04	1.10 (0.76–1.59)	1.87 (1.44–2.42)	1.56 (1.35–1.78)	3.56×10^−5^	LRFN5: FSCB
rs8060556	16	6868511	C/T	0.23	1.29 (1.04–1.59)	1.43 (1.17–1.74)	1.36 (1.14–1.69)	2.95×10^−5^	RBFOX1
rs1728400	16	86434446	C/A	0.38	1.37 (1.15–1.64)	1.25 (1.06–1.47)	1.30 (1.13–1.55)	1.40×10^−5^	LOC732275: FOXF1
rs8045253	16	86437767	T/C	0.34	1.28 (1.07–1.52)	1.32 (1.11–1.57)	1.30 (1.18–1.43)	2.82×10^−5^	LOC732275: FOXF1
rs9978224	21	41309823	G/A	0.29	1.26 (1.03–1.54)	1.38 (1.16–1.65)	1.33 (1.13–1.61)	2.61×10^−5^	TMPRSS3
rs421379	5	91275313	G/A	0.08	1.98 (1.46–2.70)	1.24 (0.91–1.68)	1.55 (1.25–1.93)	7.3×10^−5^	ARRDC3

**Table 3:** Replication of most significant associations from the discovery set meta-analysis in the replication samples.

Lead SNP	Chr	Position	Alleles	MAF	Stage-2 replicationHR (95%Confidence Interval)	Stage-2replicationp-values	All stagesmeta-analysisHR(95%Confidence Interval)	All stages meta-analysisp-value	p-value for Q-statistic	Genes
rs7441398	4	63653135	G/T	0.13	1.12 (0.90–1.39)	0.28	1.31 (1.15–1.49)	3.3×10^−5^	0.21	LPHN3: LOC644548
rs1525677	7	110302695	T/C	0.31	1.08 (0.92–1.27)	0.35	1.22 (1.10–1.34)	0.0001	0.18	IMMP2L
rs12358475	10	11848792	G/A	0.23	0.82 (0.67–1.00)	0.05	0.75 (0.67–0.84)	1.8×10^−6^	0.57	ECHDC3: C10orf47
rs2921923	10	55662089	A/G	0.49	1.03 (0.88–1.21)	0.69	1.20 (1.10–1.33)	0.0001	0.04	PCDH15
rs10777864	12	97838685	A/C	0.41	0.91 (0.77–1.07)	0.25	0.82 (0.74–0.90)	8.0×10^−5^	0.22	RMST
rs8060556	16	6868511	C/T	0.23	1.01 (0.84–1.22)	0.75	1.22 (1.09–1.36)	0.001	0.04	RBFOX1
rs1728400	16	86434446	C/A	0.38	1.16 (0.99–1.37)	0.07	1.25 (1.13–1.39)	5.6×10^−6^	0.39	LOC732275: FOXF1
rs8045253	16	86437767	T/C	0.34	1.04 (0.88–1.23)	0.65	1.17 (1.05–1.31)	0.003	0.04	LOC732275: FOXF1
rs421379	5	91275313	G/A	0.08	1.41 (1.11–1.8)	0.005	1.49 (1.27–1.75)	1.1×10^−6^	0.09	ARRDC3

Results are presented for those SNPs which remained associated in the same direction in the validation set as in the discovery set (adjusted for ER-status).

### Replication testing in POSH stage-2 samples

**Figure 2:**
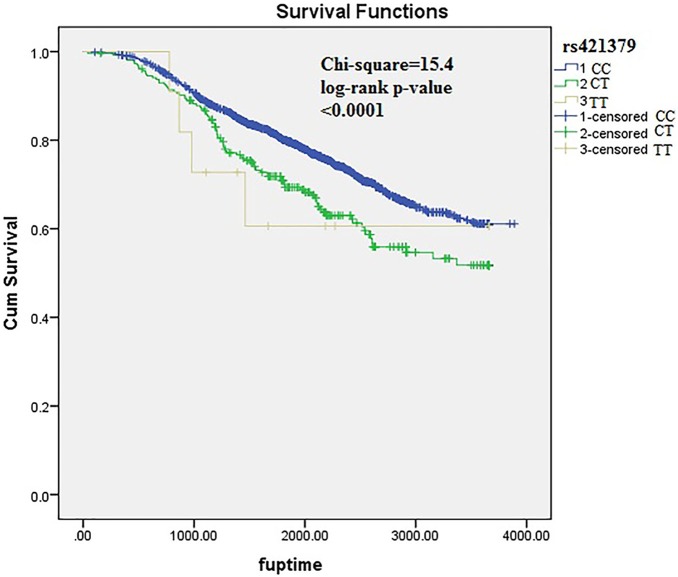
Kaplan-Meier plots depicting breast cancer related survival in response to rs421379 genotypes in pooled POSH stage-1, HEBCS and POSH stage-2 samples.

**Figure 3:**
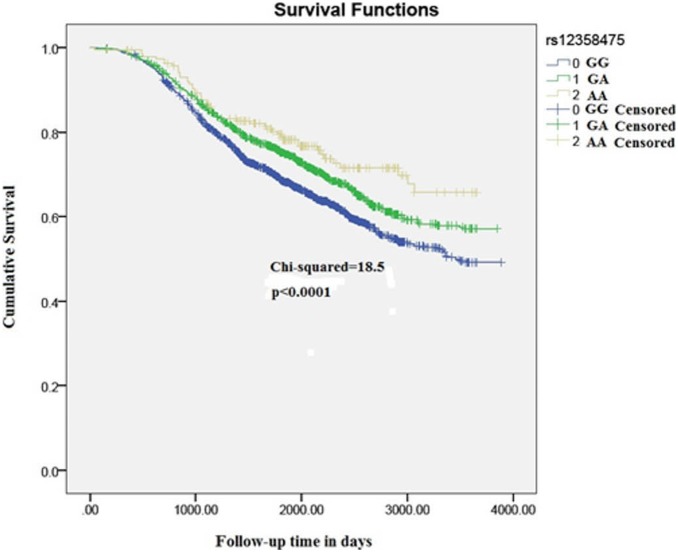
Kaplan-Meier plots depicting breast cancer related survival in response to rs12358475 genotypes in pooled POSH stage-1, HEBCS and POSH stage-2 sample.

A total of 18 SNPs with independent association signals were tested for replication in POSH stage-2 validation samples (n = 1523). One SNP demonstrated high duplicate error rate (>8%) and was excluded from analysis. Of the 18 SNPs which were formally tested for replication, two demonstrated replication signals in the validation cohort. Nine of the eighteen SNPs which were tested for replication were observed to be associated in the same direction as in the POSH and HEBCS meta-analysis. In the stage-1 and stage-2 meta-analysis the strongest association signal was observed at rs421379. The minor allele of rs421379 is found to be associated with a higher risk of breast cancer related death (HR (95% CI) = 1.49 (1.27–1.75), p = 1.1×10^−6^) (Figure 2). The p-value for Cochran’s heterogeneity test Q-statistic was not indicative of heterogeneity in meta-analysis estimate (p = 0.09). This variant was previously identified as the most significantly associated variant in a two stage GWAs for breast cancer survival in early onset cases from POSH. A weak replication signal in HEBCS allowed this SNP to be identified as the most strongly associated variant in this study too (Figure 2). The second most significant variant was located at 10p14, where the minor allele of rs12358475 was associated with protective effect on breast cancer mortality (HR (95% CI) = 0.75 (0.67–0.75), p = 1.8×10^−6^) (Figure 3). We observed another strong association with rs1728400 which is 113.6 kb upstream of the *FOXF1* locus (Table 3). In addition, the three SNPs (rs421379, rs12358475 and rs1728400) were also associated with distant disease free survival in the same direction as those observed for overall survival times, although none of these reached a genome wide level of significance (Table 4).

**Table 4:** Associations of the most significantly associated SNPs from the discovery set s with disease free survival (adjusted for ER-status).

Lead SNP	Chr	Position	Alleles	MAF	Stage −1 association(95%Confidence Interval)	HEBC associations	Stage-2 association (95%ConfidenceInterval)	All stages meta-analysis p-value	p-value for Q-statistic
rs7441398	4	63653135	G/T	0.13	1.41 (1.12–1.77)	1.11 (0.88–1.42)	1.07 (0.91–1.26)	0.01	0.15
rs1525677	7	110302695	T/C	0.31	1.21 (1.0–1.45)	1.14 (0.96–1.35)	1.05 (0.88–1.24)	0.02	0.56
rs12358475	10	11848792	G/A	0.23	0.83 (0.67–1.02)	0.78 (0.65–0.94)	0.86 (0.70–1.05)	0.001	0.77
rs2921923	10	55662089	A/G	0.49	1.42 (1.19–1.71)	1.31 (1.12–1.52)	1.08 (0.91–1.27)	3.9×10^−6^	0.08
rs10777864	12	97838685	A/C	0.41	0.80 (0.66–0.96)	0.72 (0.60–0.85)	1.07 (0.91–1.26)	0.005	0.003
rs8060556	16	6868511	C/T	0.23	1.20 (0.97–1.48)	1.30 (1.07–1.57)	0.94 (0.77–1.15)	0.03	0.06
rs1728400	16	86434446	C/A	0.38	1.27 (1.07–1.52)	1.21 (1.03–1.41)	1.17 (0.99–1.38)	5.7×10^−5^	0.81
rs8045253	16	86437767	T/C	0.34	1.27 (1.07–1.51)	1.26 (1.07–1.48)	0.90 (0.75–1.06)	0.009	0.006
rs421379	5	91275313	G/A	0.08	1.69 (1.24–2.30)	1.04 (0.76–1.43)	1.32 (0.90–1.93)	0.003	0.10

Results are presented for those SNPs which remained associated in the same direction in the replication set as in the discovery set.

### Genome wide imputation and meta-analysis

Following quality control of imputed data we had 7105428 SNPs available (maf ≥5%) in POSH stage-1 patients and 7353135 SNPs available (maf ≥5%) in the HEBCS study. In the two study meta-analysis we had close to 6.5 million SNPs available for meta-analysis. We did not identify any novel SNPs as associated with survival at p-values smaller than those observed using genotyped SNPs.

### Gene Expression variation by SNP in publically available database

We queried the Genevar 3.2.0 and SNP and CNV annotation database (scandb) to identify Cis or Tran’s eQTL effects resulting from rs12358475, rs421379 and rs1728400. No associations of rs12358475 and rs1728400 with expression of any nearby genes were noticed in Genevar. In scandb too there were no strong trans-effect associations observed with rs12358475 and rs1728400. In Scandb we observed that rs421379 had impact on expression of *ABCD1* (p = 1×10–5) and *RAB34* (p = 9×10–5).

### Univariate associations of most associated SNPs with N-stage, M-stage, T-stage and ER-status

In univariate analysis we did not observe any strong associations of rs12358475 with ER-status, N-stage, M-stage and T-stage. A nominally significant association with N-stage did not survive correction for multiple testing (Table 5). The SNP rs1728400 demonstrated weak associations with M-stage and T-stage (Table 5). No significant association of rs421379 with any of the clinical variables were observed.

**Table 5:** Associations of SNPs with nominal replication signals with clinical characteristics associated with breast cancer in a pooled set of discovery and replication cohorts.

	N-Stage			M-Stage			Estrogen Receptor status (0 = positive, 1 = negative)			T-stage		
SNP	OddsRatio	95% ConfidenceInterval	p-value	Odds Ratio	95% ConfidenceInterval	p-value	OddsRatio	95% ConfidenceInterval	p-value	OddsRatio	95%ConfidenceInterval	p-value
rs12358475	0.88	0.78–0.99	0.04	0.94	0.70–1.27	0.70	1.09	0.96–1.24	0.16	0.96	0.85–1.08	0.49
rs421379	1.06	0.83–1.35	0.65	1.25	0.93–1.67	0.14	1.09	0.89–1.34	0.39	0.98	0.77–1.25	0.88
rs1728400	1.09	0.96–1.22	0.17	1.30	0.99–1.70	0.05	1.07	0.9501.21	0.29	1.11	0.99–1.24	0.05

N-stage = metastasis to lymph node, M-stage = metastasis stage and T-stage = Tumour stage.

### Strength of association of SNPs most associated with survival in multivariate models

In pooled analysis involving the discovery and replication samples we observed a slight decrease in the strength of association at the rs421379 and rs12358475 variants. A prominent decline in association statistics at the rs1728400 variant was observed. The HR’s for rs421379 and rs12358475 after adjusting for N-stage, M-stage, ER-status, and tumour size were 1.41 (1.15–1.72), p = 0.001 and 0.85 (0.75–0.97), p = 0.01. The observed HR for rs1728400 was 1.04 (0.94–1.15) p = 0.46.

## Discussion

In this study we report a genome wide meta-analysis for identifying genetic variants associated with breast cancer related mortality. In combined meta-analysis involving 2864 individuals the strongest associations that we have identified locate to three SNPs at chromosomes 5, 10 and 16. We have previously discussed the potential biochemical pathways by which rs421379 could impact survival times [19]. It is important to note that the previous GWAs study that we had undertaken was performed exclusively in early onset cases alone. As such the findings from the current study are potentially important as these suggest a wider role for this variant in altering survival times in older breast cancer patients. We did not observe any significant effect of rs12358475 and rs421379 on clinical factors associated with breast cancer mortality suggesting that fluctuations in levels of clinical variables could be a by-product of disease rather than being driving factors.

rs12358475 is intergenic between *ECHDC3* (64 kb downstream) and C10orf47 (16 kb upstream), and 113 kb upstream of *UPF2*. *ECHDC3* encodes enoyl CoA hydratase domain containing 3 which has been described as a new inhibitor of mitochondrial fatty acid oxidation [34]. Although the clinical significance of this protein is not clear, it has been found to be differentially expressed in different breast cancer subtypes in mouse models [35]. *ECDHC3* has also been shown to be differentially expressed in acute coronary syndrome [36]. *UPG2* is involved in both mRNA nuclear export and mRNA surveillance and initiates nonsense-mediated mRNA decay (NMD) [37]. rs12358475 is predicted to disrupt a binding site for transcription factors *ETS1* and *NFAT*. ETS-1 is overexpressed in human breast cancer and this is indicative of poorer prognosis [38]–[40].

rs1728400 lies close to the *FOXF1* locus which is a putative tumour suppressor gene. This variant has previously been associated with oesophageal adenocarcinoma along with other SNPs close to rs1728400 which demonstrated even stronger associations [41]. As such if rs1728400t has a replicable impact on breast cancer prognosis then it could act via a different set of transcription factors than those activated in oesophageal carcinoma.

Although the study reported here is not the largest study undertaken for identifying common variants associated with breast cancer mortality [15], [16], it has several methodological strengths. It is the first study to Meta-analyse associations of common genetic variants with breast cancer related mortality on a genome wide level across two independent prospective studies of breast cancer patients. Further both POSH and HEBCS are prospective studies of breast cancer patients who were recruited in similar clinical settings and both cohorts have relatively high breast cancer specific mortality. As such, heterogeneity between causes of death is reduced in the meta-analysis. With respect to potential tumour phenotypic heterogeneity both studies were not selected for specific breast tumour sub-types so this remains a potential methodological problem if the effect of SNPs relates to a particular tumour sub-type or a particular modality of treatment.

It was encouraging to note that 9 off the 18 SNPs which we had marked for replication testing were associated in the same direction as in the discovery set. Furthermore 4 of the 18 SNPs which were tested for replication had previously been identified as amongst the top 50 associations in GWAs of breast cancer mortality in early onset patients. rs11723068, rs11491815, rs421379 and rs1578790 were the first, fourth, eighteenth and 20^th^ most strongly associated SNPs among the top 50 association [19].

Although previous studies have not described any SNPs as irrevocably associated with survival at genome wide levels of significance [15], [18], we attempted to test associations of the most significant SNPs from these studies. None of the 10 SNPs which Azzato et al [15] tested for replication in the SEARCH study were associated at p-values≤0.05 in the POSH and HEBCS meta-analysis results. The strongest replication signal we identified was with rs17299684 (HR = 1.15, p = 0.07). Similarly the two SNPs highlighted by Shu et al [18] as potentially associated with survival in the Chinese population, were not associated in our meta-analysis (rs3784099, HR = 0.94, P = 0.37 and rs9934948, HR = 1.09, P = 0.32). The association of SNP rs3803662 (*TOX3*), highlighted by Fasching et al [16], as potentially associated with breast cancer specific survival did not replicate in our meta-analysis (HR = 0.90, p = 0.09). The lone SNP highlighted by Azzato et al [17], as associated with survival in ER-negative patients was not available in the genome wide genotyped or imputed data, further no proxies at r^2^≥0.6 were identified based on HapMap phase 3 data. So unfortunately replication of this SNP could not be tested in our study.

Future studies with a similar ascertainment framework but with larger sample size, detailed tumour sub-type phenotyping and similar treatment modalities will be required to allow sub-type specific patient cohorts to be used for discovery and validation. A more detailed search for variants with MAF <0.05 may be necessary to fully comprehend the extent of intrinsic host genetic factors in determining breast cancer prognosis.

The main strengths of this study are the high genetic coverage achieved by using the Illumina 550 K and Illumin660 K chips in the Helsinki and POSH studies respectively. In addition we have also performed comprehensive imputation of common genetic variation (maf > = 5%) based on the LD patterns in the 1000 genomes project. We had sufficient statistical power to detect genetic variants which were associated with survival at HR≥1.23 while studying SNPs with maf ≥10%. Future studies using well annotated collaborative samples will be needed to perform sub-type specific analysis and replication to detect small effect sizes. Such a strategy has the potential to identify multiple genetic variants which are associated at HRs lower than 1.20. However a trade-off between the increases in effect sizes that may result from studying associations in specific homogeneous sub-groups may mitigate smaller sample sizes.

## Supporting Information

Checklist S1 **PRISMA Checklist.**
(DOC)
